# Maternal Obesity, Gestational Weight Gain, and Asthma in Offspring

**DOI:** 10.5888/pcd14.170196

**Published:** 2017-11-09

**Authors:** Kristen J. Polinski, Jihong Liu, Nansi S. Boghossian, Alexander C. McLain

**Affiliations:** 1 Department of Epidemiology and Biostatistics, Arnold School of Public Health, University of South Carolina, Columbia, South Carolina

## Abstract

**Introduction:**

Obesity is common among women of childbearing age; intrauterine exposure to maternal obesity or gestational weight gain may influence the development of asthma in early childhood. We examined the relationships of maternal obesity and gestational weight gain with asthma in offspring.

**Methods:**

We used data from the Early Childhood Longitudinal Study–Birth Cohort, which has a nationally representative sample of children followed from birth in 2001 through age 4 (n = 6,450). Asthma was based on parental report of a medical professional’s diagnosis. We used generalized estimating equation binomial models to compute adjusted odds ratios (ORs) of childhood asthma with maternal obesity and 4 measures of gestational weight gain.

**Results:**

Compared with children of normal-weight mothers, children of obese mothers had increased risk of asthma (adjusted OR, 1.63; 95% confidence interval [CI], 1.26–2.12) by age 4, and children born to overweight mothers had similar risk (adjusted OR, 1.25; 95% CI, 0.99–1.59). Extreme-low weight gain (<5 kg) and extreme-high weight gain (≥25 kg) were associated with increased risk of asthma; however, the following measures were not significant predictors of asthma: meeting gestational weight gain recommendations of the Institute of Medicine, total gestational weight gain, and weekly rate of weight gain in the second and third trimesters.

**Conclusion:**

Extreme-low or extreme-high gestational weight gain and maternal obesity are risk factors for early childhood asthma, further evidence of the long-term impact of intrauterine exposure on children and the need to target preconception care to improve child health indicators.

## Introduction

Approximately 7% of children receive a diagnosis of asthma by age 4 years, making asthma one of the most common chronic conditions among children (1). Childhood asthma is a leading cause of emergency department visits, hospitalizations, and missed school days (2). Asthma-management practices target symptoms and contribute to huge health care expenditures. For example, a recent nationally representative study of school-aged children in the United States found that the total annual health care expenditures burden attributable to asthma was $5.92 billion (3). Therefore, prevention and early diagnosis of asthma are particularly important in childhood.

Childhood asthma is believed to have in-utero origins (4,5); for example, a cohort study found that 40% of children with a diagnosis of asthma by age 7 had reduced airflow and bronchial responsiveness as neonates (4). Maternal weight and gestational weight gain may change the intrauterine environment and may affect the development of asthma (6). A meta-analysis of possible links between maternal body mass index (BMI), gestational weight gain, and risk of asthma in offspring concluded that maternal obesity is significantly associated with asthma in offspring; however, limited evidence was given to support associations with gestational weight gain (7).

To our knowledge, no studies have examined the association between gestational weight gain and asthma in offspring in a nationally representative sample of children in the United States. Previous studies used measures of gestational weight gain that did not account for gestational age, which can affect the amount of weight gained (ie, the shorter the pregnancy duration, the lower the weight gain). Furthermore, gestational weight gain was described inconsistently among studies (eg, as continuous data, as tertiles, as cutoffs), limiting comparability between studies.

The objective of this study was to describe the relationships of maternal obesity and gestational weight gain with risk of asthma in offspring in a multiethnic, nationally representative sample of young children in the United States.

## Methods

The Early Childhood Longitudinal Study–Birth Cohort (ECLS–B) is a longitudinal study conducted by the National Center for Education Statistics (NCES) and is designed to collect information on childhood health, development, care, and education. A nationally representative sample of children born in the United States in 2001 was selected from birth certificates, and children were followed through kindergarten entry as part of the ECLS–B (8). We used birth certificate data as well as data from assessments that took place when children were aged 9 months, 2 years, and 4 years. The weighted longitudinal response rates, which take into account response for all rounds of data collection, were reported by NCES as follows: 74.1% at 9-month collection, 69.0% at 2-year collection, and 63.1% at 4-year assessment (9). The Arnold School of Public Health is licensed to use the ECLS–B restricted-use data.

### Primary outcome

The presence of childhood asthma was assessed at each wave of data collection. In each interviewer-administered assessment, a guardian of the child was asked, “Since your child turned (*x*) years of age, has a doctor, nurse or other medical professional ever told you that your child has asthma?”

### Primary maternal exposures

In the 9-month assessment, a trained interviewer asked the mother, “How much did you weigh just before you became pregnant with [child]?” The mother was also asked to provide information on her height. These 2 questions were used to obtain data for calculation of the mother’s pre-pregnancy BMI (measured as weight in kilograms divided by height in meters squared [kg/m^2^]). Maternal BMI was classified as underweight (<18.5 kg/m^2^), normal weight (18.5–24.9 kg/m^2^), overweight (25.0–29.9 kg/m^2^) and obese (≥30.0 kg/m^2^) (10).

We obtained data on total gestational weight gain from the birth certificate, and if data on gestational weight gain were missing in birth certificates (19% of certificates), we used self-reported values. We used 4 measures of total gestational weight gain. First, we treated data on gestational weight gain as continuous data. Second, we determined a measure of weight gain adequacy based on recommendations of the Institute of Medicine (IOM) by using methods described previously (11,12). Briefly, we calculated a ratio of the observed total gestational weight gain to the expected gestational weight gain for each mother; we then categorized the ratio according to the percentage of IOM weight gain recommendations met (ie, inadequate, adequate. and excessive) based on ranges of the mother’s pre-pregnancy BMI (12). We determined expected gestational weight gain by using the following equation: Expected gestational weight gain = recommended first trimester total weight gain + [(gestational age at weight measure at or before delivery − 13 weeks) × recommended rate of gain in second and third trimesters] (11,12), where recommended total first-trimester weight gain was assumed to be 2.0 kg for underweight or normal-weight mothers,1.0 kg for overweight mothers, and 0.5 kg for obese mothers. Recommended rate of weight gain in second and third trimesters was based on the assumption that underweight, normal-weight, overweight, and obese women gain weight at the rate of 0.51 kg, 0.42 kg, 0.28 kg, and 0.22 kg per week, respectively (13). Third, we calculated the weekly rate of gestational weight gain in the second and third trimesters by dividing the estimated total weight gain in the second and third trimesters (ie, total gestational weight gain minus estimated weight gain in the first trimester per IOM guidelines) by the number of weeks in the second and third trimesters (ie, gestational age at delivery minus 13). Fourth, we divided data on gestational weight gain into 6 categories (<5 kg, 5–9 kg, 10–15 kg, 16–19 kg, 20–24 kg, and ≥25 kg) to enable comparisons between our data and data from other studies (14). We considered less than 5 kg of weight gain to be an extreme low and 25 kg be an extreme high, and we designated 10 to 15 kg as the reference level.

### Covariates

All 8 covariates were one-time measurements taken from either the birth certificate or the 9-month assessment. In addition to sociodemographic variables (mother’s race, maternal age, child’s sex), we selected, on the basis of research, the following covariates: child’s birth weight, parity, gestational age, participation in the Special Supplemental Nutrition Program for Women, Infants and Children (WIC) in the previous 12 months, and smoking during pregnancy (14–20).

### Statistical analyses

Per NCES data-use guidelines, we rounded all sample sizes to the nearest 50; thus, a sample size of 100 reflects a sample size that ranges from 75 to 124. Before any exclusions, the sample size was 10,700. After excluding twins, children with a birth weight of 500 g or less, and children whose gestational age was less than 28 weeks or more than 45 weeks, the sample was reduced to 8,550. We then removed children missing data on ECLS–B weights, which removed those who did not have a preschool assessment, reducing the sample to 6,900. Next, we removed children who had missing data on other covariates (n = 450) , leaving an analytic sample of 6,450; of these 450 children, 100 were missing data on gestational weight gain and 250 were missing data on pre-pregnancy BMI.

All analyses were adjusted for the clustered sample design and weighted to account for oversampling of particular groups and attrition (8). We tabulated baseline data on descriptive characteristics for the total analytic sample by using weighted percentages, means, and 95% confidence intervals (CIs), with inference provided by weighted χ^2^ tests (categorical variables) or *t* tests (continuous variables). With repeated asthma measurements, logistic regression models via generalized estimating equations with exchangeable correlation structure produced odds ratios (ORs) and 95% CIs for an asthma diagnosis by age 4 years. We ran 4 models, one for each measure of gestational weight gain. Each model included pre-pregnancy BMI and adjusted for all 8 covariates. We tested an interaction between each measure of gestational weight gain with pre-pregnancy BMI and child’s age at asthma diagnosis. No interactions were significant at the .10 level, and thus no interaction terms were included in any model.

As a sensitivity analysis, we applied multiple imputation methods to impute missing birth certificate data for gestational weight gain and BMI by using self-reported data and other covariates. Ten imputed data sets were analyzed and combined by using standard multiple imputation techniques (21). Briefly, we first imputed 10 data sets for continuous gestational weight gain by using PROC MI. Next, we used PROC GENMOD, using the same 8 covariates described above, to analyze each of the 10 imputed data sets. Then, PROC MIANALYZE was used to combine results for inference. All analyses were implemented in SAS version 9.3 (SAS Institute Inc).

## Results

Half of our sample were boys (51.1%), and most (84.7%) of the sample had a normal birth weight; the mean gestational age was 38.8 weeks (interquartile range, 37.5–40.0) (Table 1). At 9 months, almost half (48.9%) of the mothers had received WIC benefits in the previous 12 months, and most (89.1%) reported not smoking during pregnancy. On average, mothers in this sample gained about 12.9 kg (95% CI, 12.6–13.2) throughout pregnancy and 0.44 kg per week in the second and third trimesters (Table 2). Less than half (43.6%) of mothers exceeded IOM recommendations for gestational weight gain, 26.2% met adequate weight gain recommendations, and 30.2% gained inadequate weight during pregnancy. More than half (55.4%) of mothers had a normal pre-pregnancy BMI, and 40.5% were either overweight or obese before pregnancy.

**Table 1:** Baseline Characteristics of a Sample (n = 6,450) From the Early Childhood Longitudinal Study–Birth Cohort, United States, 2001–2005

Variables	No. (%)^a^
**Mother’s race**	
Non-Hispanic white	2,950 (60.5)
Non-Hispanic black	950 (13.0)
Hispanic	1,100 (20.8)
Non-Hispanic other	1,450 (5.7)
**Maternal age, y**	
≤19	500 (6.7)
20–24	1,600 (23.0)
25–29	1,600 (26.7)
30–34	1,600 (25.7)
≥35	1,200 (17.9)
**Parity (no. of previous deliveries) **	
0	2,700 (41.2)
1–2	3,200 (49.6)
≥3	600 (9.2)
**WIC participation in the previous 12 months**	
Yes	3,350 (48.9)
No	3,150 (51.1)
**Smoking during pregnancy**	
Yes	750 (10.9)
No	5,750 (89.1)
**Child’s birth weight, g**	
Low (<2,500)	1,050 (5.3)
Normal (2,500–3,999)	4,873 (84.7)
High (≥4,000)	550 (10.0)
**Child’s sex**	
Male	3,300 (51.1)
Female	3,150 (48.9)
**Maternal age, mean (95% CI), y**	28.4 (28.3–28.8)
**Child’s gestational age at birth, mean (95% CI), week**	38.8 (38.7–38.8)

Abbreviation: CI, confidence interval; WIC, Special Supplemental Nutrition Program for Women, Infants and Children.

a Analyses were weighted to account for attrition and oversampling of some groups. Sample sizes were unweighted and rounded to nearest 50 per data-use agreements. All values are number (percentage) unless otherwise indicated.

**Table 2:** Bivariate Associations of Maternal Gestational Weight Gain and Pre-Pregnancy BMI, by Asthma Diagnosis in Offspring by Age 4 Years, Early Childhood Longitudinal Study–Birth Cohort, United States, 2001–2005^a^

Maternal Exposures	All (n = 6,450)	Offspring Without Asthma (n = 5,350)	Offspring With Asthma (n = 1,100)	*P* Value^b^
**Total gestational weight gain, mean (95% CI), kg**	12.9 (12.6–13.2)	12.9 (12.6–13.2)	13.1 (12.5–13.7)	.50
**Rate of weight gain in the second and third trimesters, mean (95% CI), kg/week**	0.44 (0.43–0.45)	0.44 (0.43–0.45)	0.46 (0.44–0.48)	.13
**Gestational weight gain per IOM recommendations, % (no.^c^)**				
Inadequate	30.2 (2,195)	30.3	29.1	.78
Adequate	26.2 (1,662)	26.1	26.8	
Excessive	43.6 (2,615)	43.5	44.1	
**Gestational weight gain, % (no.^c^)**				
<5 kg	22.0 (1,664)	22.0	22.5	.001
5–9 kg	15.4 (1,061)	15.2	16.6	
10–15 kg	35.7 (2,217)	36.0	34.0	
16–19 kg	14.0 (805)	14.7	10.3	
20–24 kg	9.5 (536)	9.2	11.3	
≥25 kg	3.4 (189)	3.0	5.3	
**Pre-pregnancy BMI, % (no.^c^), kg/m^2^**				
Underweight (<18.5)	4.1 (360)	4.1	4.4	<.001
Normal (18.5–24.9)	55.4 (3,672)	56.4	49.8	
Overweight (25.0–29.9)	25.3 (1,480)	25.0	26.4	
Obese (≥30.0)	15.2 (960)	14.5	19.5	
**Pre-pregnancy BMI, mean (95% CI), kg/m^2^**	24.9 (24.8–25.1)	24.8 (24.6–25.0)	25.7 (25.2–26.2)	.005

Abbreviations: BMI, body mass index; CI, confidence interval; IOM, Institute of Medicine. CI: confidence interval.

a Analyses were weighted to account for attrition and oversampling of some groups. Sample sizes were unweighted and rounded to nearest 50 per data-use agreements.

b Tests compared offspring with asthma to offspring without asthma; χ^2^ test performed on categorical variables and *t* test performed on continuous variables.

c Unweighted sample sizes were reported for categorical variables in the total column only.

At 9 months, 2 years, and 4 years, 4.9%, 7.9% and 11.6% of children were ever diagnosed with asthma, respectively. Overall, 15.3% of children were ever diagnosed with asthma by age 4 years. Children with asthma were more likely to have mothers who had a BMI indicating overweight or obesity pre-pregnancy than were children without asthma. Children with asthma were more likely to have mothers with extreme-low or extreme-high gestational weight gain than were children without asthma (Table 2).

In the adjusted models (Table 3), asthma in offspring aged 9 months to 4 years of age was not significantly associated with total gestational weight gain (adjusted OR, 1.00; 95% CI, 0.98–1.02) (Model 1) or with the weekly rate of weight gain in the second and third trimesters (adjusted OR, 1.08; 95% CI, 0.69–1.71) (Model 2). The relationship comparing excessive gestational weight gain with adequate weight gain on overall asthma risk was not significantly different (adjusted OR, 0.85; 95% CI, 0.68–1.08) (Model 3). In the crude model, gaining more than 25 kg during pregnancy, compared with gaining 10 to 15 kg, was associated with increased odds of having a child with asthma (OR, 1.71, 95% CI, 1.14–2.55); this association was no longer significant after we adjusted for all covariates (adjusted OR, 1.53; 95% CI, 0.99–2.35). However, gaining less than 5 kg, compared with gaining 10 to 15 kg, was associated with increased odds of having a child with asthma after adjustment (adjusted OR, 1.56; 95% CI, 1.04–2.35).

**Table 3:** Associations of Maternal Gestational Weight Gain, Pre-Pregnancy BMI, and Asthma in Offspring by Age 4 Years From GEE Binomial Models, Early Childhood Longitudinal Study–Birth Cohort, 2001–2005

Variables	Odds Ratio (95% CI)^a^				
	Crude Model	Model 1	Model 2	Model 3	Model 4
**Total gestational weight gain, kg**	0.99 (0.97–1.01)	1.00 (0.98–1.02)	—	—	—
**Weekly rate of weight gain in the second and third trimesters, kg/week**	0.95 (0.58–1.55)	—	1.08 (0.69–1.71)	—	—
**Gestational weight gain per IOM recommendations**					
Inadequate	1.13 (0.86–1.48)	—	—	0.99 (0.76–1.32)	—
Adequate	1 [Reference]	—	—	1 [Reference]	—
Excessive	0.95 (0.76–1.19)	—	—	0.85 (0.68–1.08)	—
**Gestational weight gain, kg **					
<5	2.20 (1.50–3.20)	—	—	—	1.56 (1.04–2.35)
5–9	1.25 (0.97–1.61)	—	—	—	1.06 (0.82–1.37)
10–15	1 [Reference]	—	—	—	1 [Reference]
16–19	0.79 (0.58–1.07)	—	—	—	0.84 (0.62–1.13)
20–24	1.14 (0.83–1.55)	—	—	—	1.16 (0.85–1.59)
≥25	1.71 (1.14–2.55)	—	—	—	1.53 (0.99–2.35)
**Pre-pregnancy BMI**					
Underweight	1.18 (0.80–1.74)	1.04 (0.70–1.54)	1.04 (0.70–1.54)	—	1.07 (0.72–1.59)
Normal	1 [Reference]	1 [Reference]	1 [Reference]	—	1 [Reference]
Overweight	1.26 (0.99–1.58)	1.25 (0.99–1.59)	1.25 (0.98–1.58)	—	1.22 (0.96–1.55)
Obese	1.80 (1.40–2.31)	1.63 (1.26–2.12)	1.63 (1.26–2.11)	—	1.50 (1.15–1.95)
**Pre-pregnancy BMI (continuous)**	1.03 (1.02–1.05)	—	—	1.03 (1.01–1.05)	—

Abbreviations: — , variable not included in model; BMI, body mass index; GEE, generalized estimating equations; IOM, Institute of Medicine.

a Each model used a different measure of gestational weight gain: Model 1 (total gestational weight gain), Model 2 (rate of weight gain during second and third trimesters), Model 3 (IOM recommendations), and Model 4 (total gestational weight gain measured categorically). In addition to the adjustment for the primary exposure of interest, models 1–4 were adjusted for parity, mother’s race, mother’s age, participation in the Special Supplemental Nutrition Program for Women, Infants and Children, smoking during pregnancy, birth weight, child sex, and gestational age. Maternal BMI was included as a categorical variable in models 1, 2, and 4 and as a continuous variable in model 3.

Maternal obesity, compared with normal weight before pregnancy, was significantly associated with increased odds of having a child with asthma during the first 4 years of life (adjusted OR, 1.63; 95% CI, 1.26–2.12) (Model 1). Maternal overweight was not significantly associated (adjusted OR, 1.25; 95% CI, 0.99–1.59). Every 1.0-unit increase in BMI was associated with increased odds of asthma in children (adjusted OR, 1.03; 95% CI, 1.01–1.05) (Model 3). A sensitivity analysis that used imputed data confirmed our results from the primary analysis, with the exception of gaining less than 5 kg during pregnancy, which was no longer significant (adjusted OR, 1.30; 95% CI, 0.91–1.86).

## Discussion

Using data from a large, nationally representative birth cohort, we examined longitudinal associations of asthma in early childhood with pre-pregnancy BMI and maternal gestational weight gain. Consistent with previous studies (14,15,20), we found a modest increased risk of asthma in offspring of obese mothers compared with normal-weight mothers. We also found that pre-pregnancy overweight may have a positive but lesser effect. Consistent with our study, a meta-analysis in 2014 also showed that trends in maternal overweight and childhood risk of asthma were not significant (7).

Research is sparse on maternal gestational weight gain and asthma in offspring, although at least 2 studies that used categorical data on gestational weight gain showed increased risk of asthma (14,16). Harpsøe et al found in an adjusted model that extreme-high gestational weight gain (>25 kg), compared with a weight gain of 10 to 15 kg, was significantly associated (adjusted OR, 1.17; 95% CI, 1.02–1.33) with asthma among children aged 7 years (14). In our study population, the magnitude of association was greater for extreme-high weight gain (adjusted OR, 1.53 [95% CI, 0.99–2.35]) than it was in the study by Harpsøe et al, but the association was not significant. We found significant increased odds of asthma in offspring for low-extreme gestational weight gain (adjusted OR, 1.56 [95% CI, 1.04-2.35], but this association was not significant in the study by Harpsøe et al (adjusted OR, 1.17; 95% CI, 0.94–1.46) (14). For the other 3 measures of gestational weight gain (total gestational weight gain, meeting the 2009 IOM recommendations, and weekly rate of weight gain in the second and third trimesters), we found no significant associations with asthma in offspring.

Several possible factors may explain our results. First, BMI rather than gestational weight gain might more directly influence childhood asthma outcomes. Second, the IOM recommendations are not sensitive to the effects of weight gain that occur at crucial points in fetal development (19). Third, if a woman gains more than or less than the amount recommended by the IOM in the first trimester, then our gestational weight gain measures (ie, IOM weight gain adequacy ratio and the weekly rate of weight gain in the second and third trimesters) might have errors (22). Additionally, the IOM’s estimates of average weight gain in the first trimester were based on weight gain patterns in the 1980s, which may not accurately apply to our more recent ECLS-B cohort (22). Fourth, a meta-analysis found that childhood asthma is positively associated with children born before 32 weeks (23); however, our sample consisted of mostly full-term children (85% born at or after 37 weeks), which may further explain why our findings on gestational weight gain were not significant. Overall, better measures of gestational weight gain are needed to separate the potential effects of gestational weight gain on asthma from the effects of gestational age.

More studies are needed to further explore the underlying biological mechanisms of intrauterine exposures on asthma outcomes in offspring. For example, the influence of gestational weight gain on asthma in offspring may differ by the timing of weight gain; therefore, measuring weight gain at multiple time points during pregnancy may allow investigators to determine which patterns of weight gain might have the most effect, if any. Measures of truncal obesity, rather than BMI, may be more appropriate in capturing data on weight gain during pregnancy. Truncal obesity in pregnant women has been associated with visceral adiposity (24), which in turn could affect the mother’s metabolic profile and subsequently the outcomes of offspring. Gestational weight gain and maternal BMI are believed to influence childhood asthma through nonallergic inflammatory mechanisms; for example, high pre-pregnancy BMI and excessive gestational weight gain, particularly in the second and third trimesters, have been associated with higher levels of cord blood leptin (25,26). Furthermore, excessive weight gain during pregnancy is a strong predictor for elevated levels of tumor necrosis factor α (TNF-α) in infants, and TNF-α has been associated with asthma by age 9 years (16). Leptin and TNF-α are cell-signaling proteins known as cytokines that are involved in systemic inflammation. More specifically, leptin is a pro-inflammatory adipokine, a type of cytokine secreted from adipose tissue, and it may have pro-inflammatory effects on the child’s airways (16,27). Another potential mechanism involves the contribution of maternal obesity and excessive gestational weight gain to childhood obesity (6,28,29), which in turn may have effects on lung function and increase the risk of asthma development (30).

Strengths of our study include the use of a large, multiethnic, nationally representative population-based cohort that is generalizable to US children born in 2001. We assessed our outcome with a longitudinal design. We included 4 measures of gestational weight gain. To our knowledge, ours is the first study to include the IOM weight gain recommendations in exploring the relationship between gestational weight gain and asthma. The secondary analysis with imputed data for missing covariates replicated our findings.

Our study has limitations. First, our prevalence estimate of asthma likely captures data on related conditions (eg, wheeze), because diagnosis of asthma for children younger than 5 years is imprecise without performing spirometry; however, a similar asthma prevalence was measured in this cohort (31). As many as 50% to 80% of children with asthma develop recurring bronchitis and symptoms such as wheeze, cough, and trouble breathing before the age of 5 years (2). Second, pre-pregnancy BMI was calculated by using self-reported data on height and weight; however, maternal report is fairly consistent with direct anthropometric measurements (32). We accounted for major potential confounders; however, we did not have information about asthma severity, genetic factors, or environmental exposures such as endocrine-disrupting chemicals. Our effect estimates and CIs borderline significance, which may limit the clinical relevance of our findings; however, our findings may have implications for public health given that a high proportion of US women in our study gained weight outside of the range recommended by the IOM and a large number of women entered pregnancy overweight or obese.

Overall, our findings have important public health implications for preventing disease and promoting healthy lifestyle behaviors among mothers during pre-pregnancy. Maternal obesity is a risk factor for multiple pregnancy complications that affect both mother and child. As a modifiable risk factor, pre-pregnancy obesity should be targeted in preconception programs that promote optimal preconception weight and help women achieve and maintain a healthy weight throughout pregnancy. Although no single risk factor can entirely account for childhood asthma, such a prevention strategy may reduce the incidence of early childhood asthma in future generations.

Using data from a large nationally representative US birth cohort, our longitudinal analyses support evidence suggesting that maternal obesity and to a lesser extent overweight can affect the development of early childhood asthma. Gestational weight gain is also hypothesized to partly explain childhood asthma, and we provided evidence to support this for the 2 categories of extreme gestational weight gain. In the population we studied, excessive weight gain as defined by the IOM did not appear to be a risk factor for asthma in offspring, but future studies are needed to confirm these findings. Although a better understanding of the mechanisms of early childhood asthma is needed, our study provides evidence that intrauterine exposures to obesity may have long-term effects on children. Efforts should be made to target preconception care to help women achieve and maintain a healthy pre-pregnancy weight and promote ideal weight gain during pregnancy.
